# Junior Doctors' Experiences of Workplace Bullying, Harassment and Discrimination in Australia and Aotearoa New Zealand—A Scoping Review

**DOI:** 10.1111/ans.70323

**Published:** 2025-09-17

**Authors:** Mitchell Dwyer, Phoebe Griffin, Maryam Rouhi, Suzanne Waddingham, Lauri O′Brien, Sarah Prior

**Affiliations:** ^1^ Tasmanian School of Medicine, University of Tasmania, Medical Sciences Precinct Hobart Australia; ^2^ School of Nursing, University of Tasmania Glebe Australia; ^3^ Tasmanian School of Medicine, University of Tasmania, Rural Clinical School Cooee Australia

**Keywords:** mobbing, professional misconduct [MeSH], trainee doctors, unprofessional behavior, workplace aggression

## Abstract

**Background:**

The bullying, harassment, and discrimination (BHD) of junior doctors is associated with negative outcomes for doctors themselves and their patients. This review sought to examine literature relating to BHD among junior doctors in Australia and Aotearoa New Zealand, with a focus on definitions, prevalence rates, perpetrators, experiences/impacts, reporting, and mitigation strategies.

**Methods:**

A scoping review of peer‐reviewed and grey literature published from 2015 to 2023 was undertaken. Study participants were exclusively junior doctors in Australia and Aotearoa New Zealand. Six databases were searched during the review: PubMed, PsychInfo, Scopus, Web of Science, ProQuest Dissertations and Theses, and the Cochrane Library.

**Results:**

Eight articles were included in the review. Definitions of “bullying” emphasized the use of repeated behaviors aimed at humiliating or threatening targets. Two studies reported prevalence rates, which were in close agreement with each other. Senior doctors were identified as the most common perpetrators. Junior doctors were reluctant to use available reporting mechanisms, and there was very little evidence in support of mitigation strategies against BHD.

**Conclusion:**

Our review demonstrates that while BHD is prevalent among junior doctors, efforts to address it are hindered by methodological issues, ineffective reporting mechanisms, and a shortage of research aimed at developing and trialing mitigation strategies. Researchers seeking to examine BHD should adopt broad terms that encompass all three concepts. Interventions aimed at addressing BHD should target the attitudes and behaviors of senior doctors. The effectiveness and transparency of reporting mechanisms must be improved in order for junior doctors to fully utilize them.

## Graphical abstract

Bullying, harassment, and discrimination (BHD) are prevalent among junior doctors in Australia and New Zealand, mainly perpetrated by senior doctors. Rates of reporting are low, and mitigation strategies are scarce. Our scoping review highlights the need for broader definitions, improved reporting systems, and targeted interventions to address perpetrators' behaviors.

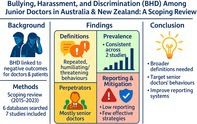

## 1 Introduction

Bullying, Harassment and Discrimination (BHD) among junior doctors in Australia and Aotearoa New Zealand remains a persistent issue [1, 2, 3]. The rigorous demands of medical training, coupled with historical norms of prolonged working hours and reliance on senior doctors for career progression, have left junior doctors vulnerable to BHD in the workplace, putting the health of patients and themselves at risk [4, 5]. Junior doctors who are in training pathways are expected to provide high‐quality patient care while developing critical skills and confidence as medical officers [6]. This period of training has traditionally been associated with long working hours, high workloads, and increased responsibility [7]. Despite the crucial role they play in healthcare delivery, junior doctors often face systemic challenges such as limited support structures, hierarchical power dynamics, and a culture of silence around BHD, which exacerbates their vulnerability to mistreatment and hinders their professional and personal growth [8, 9]. Upon completing their medical degree, junior doctors in Australia and Aotearoa New Zealand enter the workforce by completing 1 year as an “intern” or postgraduate year 1 (PGY1) doctor [10]. This internship year is typically followed by at least 1 year of working as a “resident” (PGY2+), which prepares doctors with the prerequisite skills needed to pursue specialist vocational training [10]. Their status as the least experienced doctors in the hospital system may place junior doctors at a greater risk of experiencing workplace BHD [11]. Moreover, unlike their colleagues who have commenced specialist training, junior doctors undertaking prevocational training do not have avenues within specialty colleges to report negative workplace behaviors and must rely on internal processes within their place of employment [12].

International studies have shown that BHD contributes to psychological distress in junior doctors [13], which may affect their training and compromise the quality of care they provide to patients [4, 5]. Burnout, job dissatisfaction, reduced job performance, medical errors, and higher rates of suicide ideation have all been linked to workplace bullying of junior doctors [5, 14]. Age and postgraduate experience were reported to be associated with exposure to bullying in junior doctors [11], with traditional professional hierarchical structures and “tough love” in medical training remaining an expectation within this field. Similarly, in recent studies, junior doctors report experiencing racism, sexual discrimination, and not feeling physically safe during their training [7, 15]. The impact of discrimination, in particular racism, has been reported to include unequal access to training opportunities, research, and career progression opportunities for individuals, as well as eroding health systems and economies from reduced capacity and increased staff turnover [16].

Research has previously focused on the prevalence, risk factors, and outcomes of bullying, including harassment and discrimination in junior doctors generally [17]. Fewer studies have explored the current in‐depth experiences of junior doctors or the processes around reporting, disclosing, and addressing BHD. Despite most (85%) trainees within Australian health services indicating they know how to report incidents of BHD, only 1 in 3 of those who experienced BHD reported the incident [1]. There is also low satisfaction with the handling of reported incidents in Australia [18]. The experiences and perceptions of junior doctors in Australia and Aotearoa New Zealand are crucial to inform and mitigate these challenges within the medical workforce. This scoping review aims to explore existing literature on the experiences and perceptions of Australian and Aotearoa New Zealand doctors in prevocational training regarding workplace BHD. We have chosen to focus our review on doctors undertaking pre‐vocational training as this group may be more vulnerable to BHD and has access to limited reporting mechanisms. Specifically, this review sought information about junior doctor BHD in relation to:

1. Definitions
2. Prevalence
3. Perpetrators
4. Experiences and impacts
5. Reporting
6. Mitigation strategies

## 2 Materials and Methods

This scoping review used a systematic search strategy guided by Preferred Reporting Items for Systematic Reviews and Meta‐Analyses Extension for Scoping Reviews (PRISMA‐ScR) [19]. A copy of the PRISMA‐ScR checklist can be found in the Supporting Information section. A modified Population, Interest, Context (PICo) review protocol criteria [20] was formulated by the authors to develop key concepts for searching databases and determining eligibility criteria (Table 1). As this scoping review was exploratory without a specific focus on outcomes, the concept of “outcomes” in PICO was not used to develop key concepts for searching. A copy of this protocol is available from the authors upon request.

**TABLE 1:** Modified PICO criteria.

Population	Interest	Context
Junior doctors	Bullying	Australia
	Harassment	New Zealand
		Acute setting
	Discrimination	Subacute setting
		General practice

### 2.1 Eligibility Criteria

The review considered quantitative, qualitative, and mixed method studies, with a full text written in English from 2015 to 2024. Participants were exclusively junior doctors in Australia and Aotearoa New Zealand. Articles concentrating on more senior doctors or other medical professionals such as registrars, senior medical officers, or consultants were excluded, particularly those unable to distinguish findings specific to junior doctors from the broader participant group.

### 2.2 Search Strategy

An initial literature search was conducted to uncover any pertinent Australian and Aotearoa New Zealand studies focused on junior doctor experiences of bullying, harassment, and/or discrimination. Frequently used vocabulary was derived from article titles, abstracts, and keywords, and consultation with a research librarian to develop key terms for the systematic search. Following team consensus on the finalized search terms, six databases were searched: PubMed, PsychInfo, Scopus, Web of Science, ProQuest Dissertations and Theses, and the Cochrane Library. The reference lists of identified articles were also scanned for relevant articles, and Google Scholar was hand searched for any further literature.

The following search terms were used: “junior doctor” OR “intern” OR “house officer” OR “foundation doctor” OR “trainee doctor” OR “doctors in training” OR “resident” OR “junior medical officer*” OR “newly qualified doctor” AND “mobbing” OR “bullying” OR “victimization” OR “victimization” OR “harassment” OR “emotional abuse” OR “aggression” AND “hospital” OR “healthcare” OR “health service” OR “acute” OR “subacute” OR “general practice” OR “junior physician” OR “junior practitioner” AND “Australia” OR “New Zealand” OR “Aotearoa”. Details of the search strategy used within the PubMed database can be found in Appendix A. All results were imported into the Covidence software (Veritas Health Innovation, 2023). Duplicate articles were automatically removed by Covidence. All authors screened the titles and abstracts independently according to the inclusion and exclusion criteria. The full text of papers included in the review was uploaded and assessed by two research team members. All conflicts were discussed by the team and a consensus decision was reached.

### 2.3 Data Extraction and Synthesis

Data were extracted by 3 team members (M.R., P.G., and S.W.) using a purpose‐designed data extraction tool. This included authors' names, publication date, aims of the study, methodology, method, setting, participants, data analysis, outcomes/findings, definitions, perpetrators, and strategies. Basic deductive qualitative content analysis was used to map data to each category of interest and synthesize the findings [21]. Microsoft Excel 365 was used to organize the data.

## 3 Results

**FIGURE 1:**
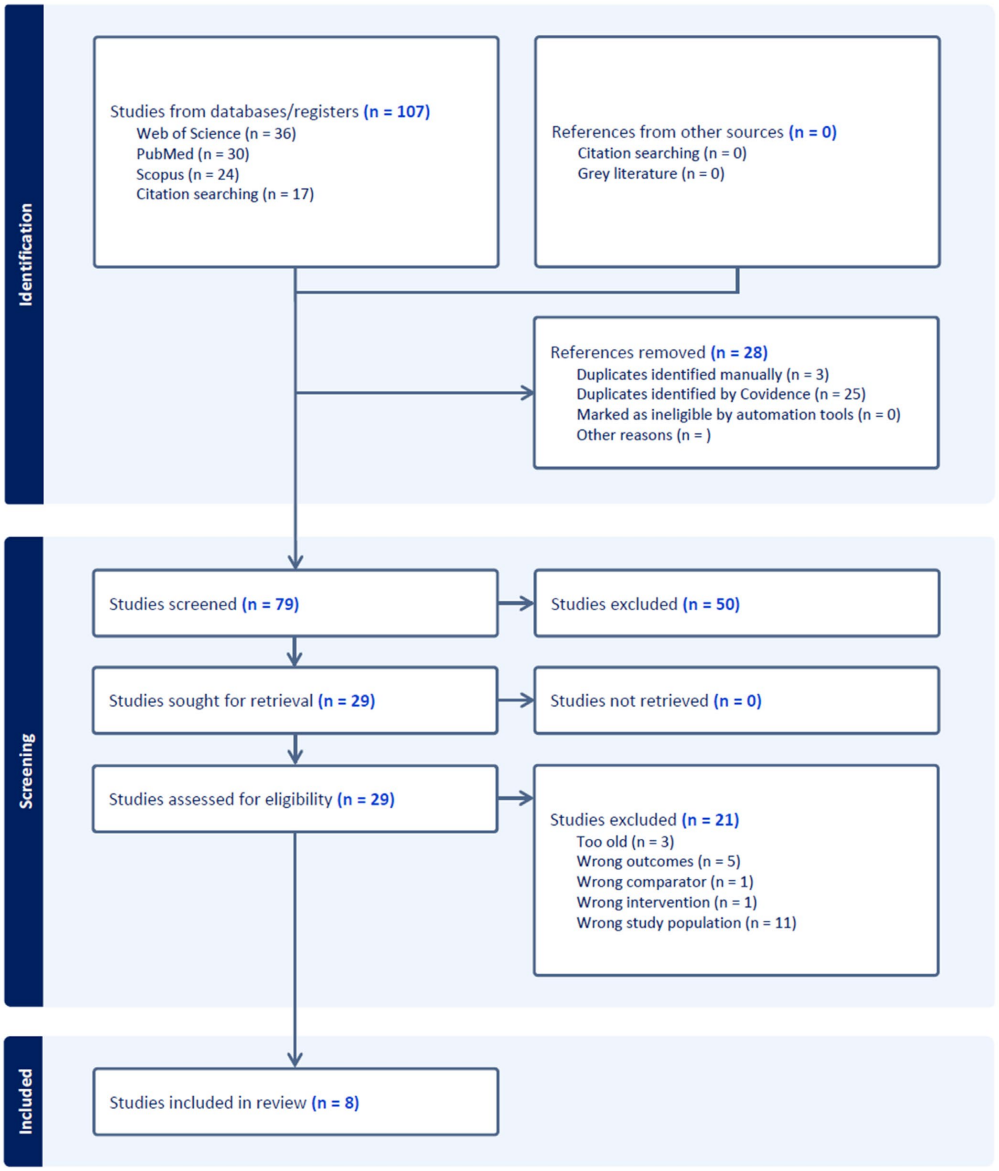
Flow diagram of the screening process.

We illustrate the flow of articles through the screening process in Figure 1 below. A total of eight papers met the eligibility criteria and were subsequently extracted and reviewed.

The final eight articles for review (Table 2) included one government report [22], three qualitative studies [23, 24, 25], three cross‐sectional survey‐based studies [26, 27, 28], and one scoping review [29]. While all articles included doctors in prevocational training, only three focused exclusively on this cohort [24, 26, 27]. Other studies examined the experiences of health care workers in general [22, 23] or included both prevocational and vocational trainees [25, 28, 29]. One study was based in Victoria [22], two in New South Wales/Australian Capital Territory [26, 27], one in Queensland [24], and two were Australia‐wide [23, 28, 29]. Only one study encompassed both Aotearoa New Zealand and Australia [28]. No articles examined all of BHD. Of those that did look at aspects of BHD, three reported solely on bullying [23, 26, 29], while others explored bullying and harassment [22, 27], bullying and racism [28], and sexual harassment and abuse [25]. None of the studies explicitly examined discrimination. An overview of how included articles related to each topic of interest is provided in Table 3, with basic content analysis following.

**TABLE 2:** Summary of included studies.

Reference, Country	Study aim/s	Design	Key findings relating to BHD
Ananda‐Rajah, et al. 2021 Australia	To examine HCWs' reported challenges they have faced during the COVID‐19 pandemic.	Qualitative cross‐sectional	PPE use during COVID‐19 was reported to have exacerbated BHD.
		331 Doctors, including Junior doctors	
Forbes, et al. 2019 Australia	To explore factors associated with the psychological well‐being of junior doctors.	Qualitative exploratory	Bullying and harassment emerged as a key theme affecting junior doctor health and well‐being. Bullying and harassment were perceived as being an integral part of medicine and led to feelings of disillusionment, inadequacy and belittlement. Reporting was avoided due to perceived threat of reprisal and concerns over career progression.
		15 Junior doctors (PGY1 and PGY2)	
Ganes and Sunder 2024 Australia	To explore the impact of medical training on the mental health of Australian medical students and doctors in training	Scoping review	Workplace bullying was associated with higher rates of psychological distress among junior doctors. Workplace bullying by colleagues and patients was identified as a commonly reported barrier to psychological well being. Studies exploring harassment and discrimination were less well represented
Lau, et al. 2017 Australia	To determine the prevalence of psychological distress in Australian junior medical officers (JMO) and investigate the determinants associated with psychological distress over a 3‐year (2014–2016) period.	Quantitative cross‐sectional	Compared to those who experience daily bullying, participants who never experienced bullying had lower odds of a high K10 (likely to have a moderate or severe mental disorder).
		1085 Junior doctor respondents (mainly PGY1 and 2, with a small number of PGY3)	
Llewellyn, et al. 2019 Australia	To describe rates of exposure to bullying and sexual harassment in junior doctors in first‐ or second‐year prevocational medical training (PGY1 or PGY2 respectively) positions in New South Wales (NSW) and the Australian Capital Territory (ACT); to explore the types of actions taken in response; and explore the experiences of junior doctors of the reporting process.	Mixed method, cross‐sectional	Over half of respondents reported being bullied with 16%–19% reporting being sexually harassed. Senior doctors were most frequently identified as the perpetrator. Reasons for not acting in response to bullying and harassment included workplace normalization of negative behaviors, fear of reprisal, lack of knowledge or confidence in the reporting process.
		814 Junior doctors (PGY1 and PGY2)	
Petrie, et al. 2021b Australia and New Zealand	To examine workplace stressors reported by junior doctors and identify variables associated with adverse mental health outcomes.	Quantitative cross‐sectional	Experiences of bullying and racism were associated with Common Mental Disorder. Bullying was also associated with suicide ideation.
		3053 Junior doctors (pre‐vocational and vocational trainees)	
Stone, et al. 2019 Australia	To identify, using personal accounts, the impact on doctors of sexual harassment and assault by doctors in the workplace.	Qualitative narrative	Participants described their personal or structural vulnerability to sexual abuse, and of discounting the experience. Reporting involved a risk of harm both professionally and personally. Those who reported the abuse faced exposure, feelings of shame and the withdrawal of colleagues. In the aftermath, participants struggled to re‐assert their professional identities. Most had a long period of absence from the workplace.
		6 Junior doctors	
Victorian Auditor‐General 2016 Australia	To examine whether public health services and Ambulance Victoria (AV) are effectively managing the risk of bullying and harassment in the workplace. To also examine the roles of the Department of Health & Human Services (DHHS), WorkSafe and the Victorian Public Sector Commission (VPSC), in providing sector‐wide leadership, support and guidance.	Pragmatic multi‐method audit/review	Participants reported that there was no point in reporting instances of bullying or harassment, and that health services fail to hold perpetrators to account. Participants described a high degree of acceptance of negative behaviors. Some reported having poor knowledge of how to report BHD and/or where to go to for support.
		21 Junior doctors participated in focus groups	

**TABLE 3:** Overview of content related to BHD in included studies.

	Ananda‐Rajah et al., 2021	Forbes et al., 2019	Ganes and Sunder 2024	Lau et al., 2017	Llewellyn et al., 2019	Petrie et al., 2021	Stone et al., 2019	Victorian Auditor‐General, 2016
Definition					B, SH		SH, SA	B, H
Prevalence					B, SH			
Perpetrator		B, H			B		SH, SA	
Experiences and impacts	B	B, H	B	B	B, SH	B, R	SH, SA	B, H
Reporting		B, H			B, SH		SH, SA	B, H
Mitigation strategies							SH, SA	B, H^a^

Abbreviations: B, bullying; D, discrimination; H, harassment; *R*, racism; SA, sexual abuse; SH, sexual harassment.

^a^ Strategies not specific to junior doctor cohort but healthcare workers in general.

### 3.1 Definitions

Only three of the included studies provided definitions for bullying, harassment, and/or discrimination (depending on the phenomena of interest). Two studies defined bullying. Common elements of both definitions included repeated behaviors with the outcome of humiliating or threatening the target [22, 27]. Definitions differed in whether bullying could be unintentional [22] and in their effects on targets (coercion, intimidation, dominance) [22]. Sexual harassment was defined in two studies but quite differently. Stone and colleagues [25] (835) defined sexual harassment as “improper or unwelcome conduct that could reasonably be perceived to cause offense or humiliation to another person” and as a form of sexual abuse. Llewellyn et al. [27] (329) defined sexual harassment as the “the making of unwanted sexual advances or obscene remarks”. A definition of harassment was provided in only one study [22]. Harassment was described as occurring when “a person or a group is treated less favorably than another person or group because of a particular characteristic—such as ethnic origin, gender, age, disability or religion” [22] (2). Provided definitions were largely drawn from national legal definitions [22, 25] but also the World Health Organization in the case of one study [25]. Llewellyn et al. [27] did not identify a source for their definition.

### 3.2 Prevalence

The prevalence of experiences of bullying among junior doctors in NSW and ACT was reported as 54.3% and 57.5% in 2015 and 2016, respectively [27]. While prevalence was not explicitly reported in Lau et al. [26], only 43.8% of junior doctor participants responded as never having experienced bullying. Between 16 and 19% of junior doctors in NSW and ACT reported being sexually harassed [27]. Experiences of bullying and sexual harassment were significantly more prevalent among female junior doctors than male [27]. None of the included studies reported on the prevalence of discrimination or other forms of harassment, such as racism.

### 3.3 Perpetrators

Three studies identified the reported perpetrators of BHD behaviors. The most reported perpetrators of bullying were senior doctors, often in a supervisory capacity [24, 27]. Other healthcare staff, including nurses and managers, registrars, peers, and patients were also identified as perpetrators of bullying [24, 27]. In their small qualitative study, Stone et al. [25] reported that consultants, clinical supervisors, and registrars within the same team were identified as perpetrators of sexual harassment and abuse. In another qualitative study, junior doctors described experiences of being harassed, including both racially and sexually, most often by patients [24], and that this was largely normalized. Again, female participants appeared to be disproportionately targeted [24].

### 3.4 Experiences and Impacts

All included studies described how BHD was experienced by junior doctors and/or how it affected them. Two studies reported that junior doctors perceived BHD as an accepted and normalized, even integral, part of medicine [22, 24]. Despite being normalized, serious effects of BHD were reported in most studies. Experiences of BHD led to junior doctors feeling disillusioned, inadequate, vulnerable, and belittled [24, 25]. Ananda‐Rajah et al. [23] described how the COVID‐19 pandemic may have further contributed to BHD as personal protective equipment was inadequate, in limited supply, or deemed unnecessary. Trainee doctors (as well as other healthcare workers) felt their safety was compromised, and they were chastised for speaking up [23]. Longer term, BHD affected junior doctors' mental health [24, 25, 29] and their careers, with some leaving medicine [25]. Three included studies quantified the association between experiences of BHD and mental health impacts. Junior doctors who reported being bullied were found to have higher levels of psychological distress than those not bullied [27] and have a greater likelihood of common mental disorder and suicidal ideation [26, 28]. Experiences of racism were also found to be independently associated with common mental disorder in junior doctors [28].

### 3.5 Reporting

The rate at which junior doctors report incidents of BHD was quantified in very few of the included studies. In the survey‐based study by Llewellyn et al. [27], just 82 of 834 (10%) of participants indicated they had reported an incidence of BHD, suggesting reporting rates are relatively low given reported BHD prevalence. However, four studies explored barriers to reporting [22, 24, 25, 27]. Commonly reported barriers included fear of retribution or reprisal, damage to reputations and careers, poor understanding or confidence in reporting processes, and being discouraged from reporting by colleagues [22, 24, 25, 27]. The normalization of BHD was also seen as a barrier to reporting [24], as was becoming disengaged from the organization [27]. Reporting processes were perceived by junior doctors as lacking in transparency, ineffective, and potentially harmful, with insufficient avenues of action and redress [22, 24, 27].

Those who reported BHD did so by escalating their complaint to senior supervisors, with fewer sharing their experiences with peers and directly confronting the perpetrator [27]. Participants in the study by Llewellyn et al. [27] who indicated they experienced effective reporting processes described feeling supported through the process by senior staff and speaking directly with the perpetrator. Participants who experienced ineffective or harmful reporting processes felt dismissed and lost trust in the process and organization, with ineffective solutions and logistical barriers also nominated as barriers to effective reporting processes [27]. Stone et al. [25] found that participants who reported their experiences of sexual harassment and abuse felt exposed, which led to feelings of shame. Some participants described colleagues withdrawing from them after making a complaint, while others mentioned being well supported by colleagues [25].

### 3.6 Mitigation Strategies

Two of the included studies described strategies for preventing or responding to workplace BHD. The Victorian Auditor‐General's Report [22], which examined BHD in the broader Victorian public health sector, found that current policies and procedures did not act as effective controls for reducing inappropriate behaviors, including bullying and harassment. Training and education efforts to combat inappropriate behaviors, such as bullying and harassment, were described as inadequate in the audited agencies. Available training was found to be limited, sporadic, and often not mandatory for all staff levels. Managers lacked consistent training in prevention and response skills, hindering their ability to foster a positive workplace culture. Additionally, early intervention strategies were found not to be adequately supported by training. Audited agencies were reported to lack clear, fully evaluated policies [22]. Recommendations made by the report included early intervention and improving policies and procedures to ensure they were transparent, well‐scoped, and robust. Other recommendations included training and education for managers and staff as well as building a positive and respectful workplace culture through campaigns, organisation‐wide programs, and improved leadership [22]. Stone et al. [25] argued that mechanisms for managing sexual abuse, including harassment, should focus on support, reducing power differentials within the medical workforce, and restorative justice.

## 4 Discussion

This review mapped existing literature on the experiences and perceptions of Australian and Aotearoa New Zealand doctors in prevocational training regarding workplace BHD. Eight articles met inclusion criteria and were included in the review. Three included definitions of bullying, harassment, and/or discrimination; one study explicitly reported on prevalence; three studies identified perpetrators; all eight described the experiences and impacts of BDH on prevocational trainees; four studies examined reporting; and two discussed mitigation strategies. Within the studies that provided definitions of bullying, the common elements of these definitions were: (a) the presence of repeated behaviors with (b) the outcome of humiliating or threatening the target. This finding is consistent with seminal literature on the topic, where bullying is defined as “…repeated and enduring aggressive behaviors that are intended to be hostile and/or that reasonably may be perceived as hostile by the recipient” [30] (11). The absence of definitions in the remaining studies may be due to a reluctance on the part of researchers to commit to specific definitions of BHD, as there are often discrepancies found between the definitions of bullying adopted by researchers and their participants' understanding of bullying [31]. Indeed, one meta‐analysis found that subjects were more likely to report instances of self‐labeled bullying as opposed to instances of bullying that conformed to a definition provided by the researchers [32]. Hence, it may be preferable for researchers to allow their participants to determine what experiences come under broad umbrella terms such as “bullying”, in the interest of capturing as many insights as possible.

Prevalence rates of BHD were addressed in two of the included studies [26, 27] and this was limited to the estimated prevalence of bullying only. These rates were in close agreement with each other, with 54%–56% of junior doctors estimated to have experienced bullying. This finding was also consistent with the prevalence of bullying reported in a meta‐analysis of 13 international studies, which stood at 51% [33]. Remarkably, however, the rates found in this review are notably higher than those found in the wider body of literature on workplace bullying. In their meta‐analysis of 102 prevalence estimates of bullying in a range of industries (*N* = 130 973), Nielsen et al. [32] reported an overall estimate of 14.6%, with very few of the reviewed studies reporting levels comparable to that of Llewellyn et al. [27] and Lau et al. [26]. Of course, prevalence rates of BHD are strongly influenced by how the concept is defined, which, as noted above, varies both between individuals and researchers. The absence of prevalence rates for harassment and discrimination may be partly due to a conceptual overlap they share with bullying, with the latter seemingly being the more commonly used term. For instance, “bullying” is portrayed in scientific and legal literature as being: (a) a form of discrimination [34], (b) a term that may be used interchangeably with “harassment” and “mobbing” [30], and (c) a precursor of “harassment”, which only occurs once the bullying behavior is focused on certain attributes of the victim (e.g., their behavior or personality) [35]. It is for this reason why some theorists have advocated for the use of umbrella terms such as “workplace aggression” [36] and “unprofessional behavior” [37]. As noted by Einarsen et al. [30], there may be little value in distinguishing between bullying and related concepts if each of them is to be managed in a similar way.

Reported perpetrators of BHD were most commonly senior doctors who were the supervisors of the victim, followed by managers and nursing staff. This pattern of BHD being largely perpetrated by senior doctors is consistent with one Australian study, which pre‐dates the inclusion period for the current review [38]. This finding is also not surprising, given that the presence of a power differential is often regarded to be an essential component of bullying [39, 40]. All included studies addressed the experiences and/or impacts of victims in one form or another. These included detrimental effects on junior doctors' mental health as well as feelings of disillusionment, inadequacy, vulnerability, and belittlement. It is interesting to note that “being overworked” did not feature prominently, given that a previous systematic review found this to be the most reported form of bullying within teaching hospitals [17]. Of the studies that discussed the reporting of BHD, it was clear that very few junior doctors felt confident in reporting systems and processes. Our review demonstrated that there are several main factors influencing the decision to report, including effectiveness, transparency, and support. A systematic literature review focused on surgical residents in the United States of America suggested that reporting behavior is generally outcome dependent, with 56% of residents who reported BHD having an ongoing negative experience and 51% of those who did not report BHD fearing retaliation [41], which is consistent with the Australian and Aotearoa New Zealand context.

Only two of the included studies addressed mitigation strategies in some manner, and neither proposed any strategies which had, at the time of publication, been proven effective. As noted by Escartin [42], the body of research on interventions for workplace bullying and their effectiveness had traditionally lagged behind studies describing the occurrence of bullying. While beyond the scope of this review, a recently proposed framework for effective bullying, discrimination, and harassment handling aims to promote consistency in how BHD is prevented, responded to, and monitored across the Australian healthcare system. The proposed framework emphasizes the role of specialty colleges and employers, advocates for regular education, and includes the establishment of an independent Australia‐wide complaints investigation body [43]. However, stronger reporting mechanisms and education alone are unlikely to prevent junior doctors from experiencing workplace BDH. In recent times, prominent theorists Zapf and Vartia [44] created a taxonomy of interventions, describing a range of strategies at the individual, organizational, and societal levels, which are focused on the primary, secondary, and tertiary prevention of bullying. The authors note that future researchers should continue to experiment with new interventions, employing high‐quality study designs.

This review has some limitations that must be acknowledged. The scope of our review was restricted to junior doctors working in Australia and Aotearoa New Zealand. It is therefore likely that a more comprehensive understanding of the topic could be obtained by using a worldwide focus. Future research in this area should also work toward a consensus on which umbrella term (or terms) are most appropriate for describing BHD in its entirety. By having a widely adopted operational definition, there would be less risk of researchers missing out on valuable insights from participants due to semantic nuances; it may also facilitate collaboration and learning between researchers who are experimenting with interventions to address BHD. Secondly, some of the included studies' participants included doctors who had commenced their vocational training. For this reason, we cannot claim that our review's findings pertain to prevocational doctors exclusively. Lastly, our review included one scoping review, which largely reviewed primary studies that were also included in this review. This may have introduced some duplication in our findings.

## 5 Conclusions

Our review, the first to have examined literature relating to BHD among junior doctors in Australia and Aotearoa New Zealand, demonstrates that BHD remains a prominent issue, which is exacerbated by reporting mechanisms that are often ineffective. Progress in this field of research appears to be hampered by methodological issues associated with measuring BHD, along with the tendency of researchers to describe the occurrence of BHD, rather than experiment with novel mitigation strategies. The findings of this review add weight to the notion that the concepts of bullying, harassment, and discrimination overlap with each other, and that parties seeking to measure and address BHD should adopt broader terms to encapsulate all three concepts. Reported prevalence rates of BHD vary depending on the definition of BHD used and should therefore be interpreted with due caution. Senior doctors are the most common perpetrators of BHD against junior doctors, and hence, interventions seeking to address BHD should target senior doctors' behavior as a priority. Junior doctors are not confident in the mechanisms available to report BHD and would benefit from processes that are more effective and transparent, along with more support to make reports in the first instance.

## Author Contributions

**Mitchell Dwyer:** conceptualization, methodology, writing – original draft, writing – review and editing. **Phoebe Griffin:** conceptualization, methodology, writing – original draft, writing – review and editing. **Maryam Rouhi:** conceptualization, methodology, writing – review and editing. **Suzanne Waddingham:** conceptualization, methodology, writing – review and editing. **Lauri O′Brien:** conceptualization, methodology, writing – review and editing. **Sarah Prior:** conceptualization, methodology, writing – review and editing.

## Disclosure

As noted above, the authors were successful in obtaining a grant from the PMCT to conduct this body of work, which also includes a separate qualitative study (in progress). Given the nature of the topic and the funding body, we do not believe that this relationship poses a conflict of interest.

## Conflicts of Interest

The authors declare no conflicts of interest.

## Data Availability

The data that support the findings of this study are available from the corresponding author upon reasonable request.

## Appendix A PubMed Search Strategy

1. “junior doctor”
2. “intern”
3. “house officer”
4. “foundation doctor”
5. “trainee doctor”
6. “doctors in training”
7. “resident”
8. “junior medical officer*”
9. “newly qualified doctor”
10. “junior physician”
11. “junior practitioner”
12. #1 OR #2 OR #3 OR #4 OR #5 OR #6 OR #7 OR #8 OR #9 OR #10 OR #11
13. “mobbing”
14. “bullying”
15. “victimization”
16. “victimization”
17. “harassment”
18. “emotional abuse”
19. “aggression”
20. #13 OR #14 OR #15 OR #16 OR #17 OR #18 OR #19
21. “hospital”
22. “healthcare”
23. “health service”
24. “acute”
25. “subacute”
26. “general practice”
27. #21 OR #22 OR #23 OR #24 OR #25 OR #26
28. “Australia”
29. “New Zealand”
30. “Aotearoa”
31. #28 OR #29 OR #30
32. #12 AND #20 AND #27
